# Purification of Modified Therapeutic Proteins Available on the Market: An Analysis of Chromatography-Based Strategies

**DOI:** 10.3389/fbioe.2021.717326

**Published:** 2021-08-20

**Authors:** Calef Sánchez-Trasviña, Miguel Flores-Gatica, Daniela Enriquez-Ochoa, Marco Rito-Palomares, Karla Mayolo-Deloisa

**Affiliations:** ^1^ Tecnologico de Monterrey, Escuela de Ingeniería y Ciencias, Centro de Biotecnología-FEMSA, Monterrey, Mexico; ^2^ Tecnologico de Monterrey, Escuela de Medicina y Ciencias de la Salud, Monterrey, Mexico

**Keywords:** chromatography, purification, PEGylation, lipidation, Fc-fusion, biopharmaceuticals, protein modification

## Abstract

Proteins, which have inherent biorecognition properties, have long been used as therapeutic agents for the treatment of a wide variety of clinical indications. Protein modification through covalent attachment to different moieties improves the therapeutic’s pharmacokinetic properties, affinity, stability, confers protection against proteolytic degradation, and increases circulation half-life. Nowadays, several modified therapeutic proteins, including PEGylated, Fc-fused, lipidated, albumin-fused, and glycosylated proteins have obtained regulatory approval for commercialization. During its manufacturing, the purification steps of the therapeutic agent are decisive to ensure the quality, effectiveness, potency, and safety of the final product. Due to the robustness, selectivity, and high resolution of chromatographic methods, these are recognized as the gold standard in the downstream processing of therapeutic proteins. Moreover, depending on the modification strategy, the protein will suffer different physicochemical changes, which must be considered to define a purification approach. This review aims to deeply analyze the purification methods employed for modified therapeutic proteins that are currently available on the market, to understand why the selected strategies were successful. Emphasis is placed on chromatographic methods since they govern the purification processes within the pharmaceutical industry. Furthermore, to discuss how the modification type strongly influences the purification strategy, the purification processes of three different modified versions of coagulation factor IX are contrasted.

## Introduction

Within the pharmaceutical industry, biopharmaceuticals have become a continuously growing category in recent years (Papathanasiou and Kontoravdi, 2020). In the last 19 years, more than 300 biopharmaceuticals were approved (Tihanyi and Nyitray, 2021). In 2018, biopharmaceuticals accounted for almost 40% of the total drug research and development pipeline worldwide; while global sales reached nearly USD 228 billion in 2016 (Moorkens et al., 2017; Lloyd, 2018). Among biopharmaceuticals, therapeutic proteins have been recognized as the most important biologicals in terms of clinical utility (Dimitrov, 2012). From 2014 to 2018, 68 monoclonal antibodies, 23 hormones, 16 clotting factors, and nine enzymes were approved in the United States and the European Union (Walsh, 2018). In 2020, the global sales of therapeutic proteins reached USD 90.53 billion, and it is expected to reach more than USD 155 billion in 2025 (Research and Markets, 2021).

Therapeutic proteins (including native, recombinant proteins, and monoclonal antibodies) have been used successfully for a wide variety of treatments including the restoration of native biomolecules activity, integration of non-present proteins, control of metabolic pathways, and even cancer (Pelegri-O´Day et al., 2014). From a therapeutic perspective, they offer superior advantages over conventional drugs, such as being highly specific and providing complex functions, having a high biological activity, and less risk of side effects (Pisal et al., 2010). However, there are several limitations associated with their use (excluding to monoclonal antibodies), such as short half-life within the body, low solubility, physicochemical instability, susceptibility to proteolytic degradation, and immunogenicity (Pfister and Morbidelli, 2014). Protein modification has emerged as a strategy to overcome these challenges.

**FIGURE 1:**
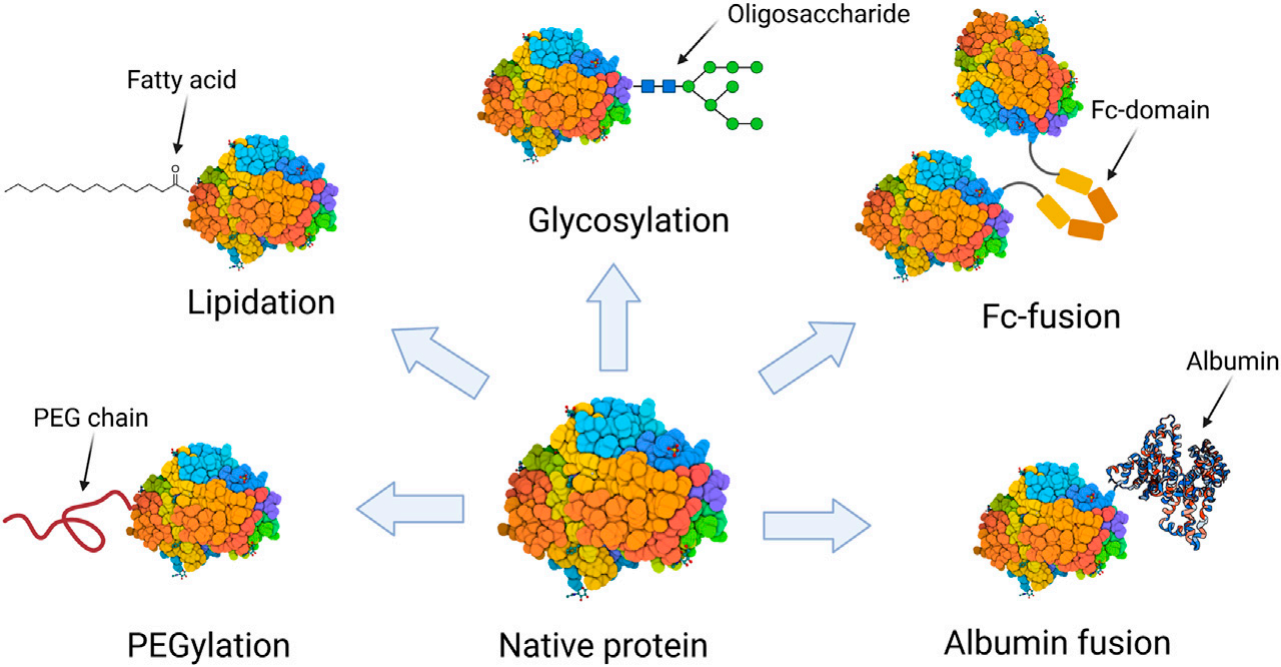
Schematic representation of the protein modification approaches that are present by at least one biopharmaceutical on the market.

Protein modification involves the conjugation or fusion of a protein to a specific moiety, such as polymers, lipids, glycosides, peptides, protein domains or another proteins (Strohl, 2015; Veronese and Pasut, 2007). Despite that many protein modification approaches using different moieties have been proposed, just a few of them have become widely adopted for therapeutic proteins (Figure 1). Modification with polyethylene glycol (PEG) moieties has undoubtedly become the most used. Since the approval of the first PEGylated protein in 1990, Adagen® (a PEG-conjugate adenosine deaminase), 29 PEGylated protein biopharmaceuticals have been approved and are currently available on the market. Another commonly used approach to improve therapeutic proteins’ pharmacokinetics is the fusion to the Fc domain of immunoglobulin G (IgG). In 1998, Enbrel® became the first Fc-fusion protein to be available on the market and thence 12 Fc-fusion proteins have obtained regulatory approval (Jafari et al., 2017). Other protein modification approaches, such as lipidation, albumin-fusion, and glycosylation have gained increasing interest over the past years. Nevertheless, they still have a reduced presence on the market. To date, there are only six lipidated proteins, one glycosylated protein, and one albumin-fusion protein approved for therapeutic use in human subjects (van Witteloostuijn et al., 2016; Lagassé et al., 2017).

To obtain regulatory approval and successfully reach the market, therapeutic proteins must be manufactured under conditions that ensure their safety and efficacy. This is not a trivial process. Therapeutic proteins are generally produced using living cells or microorganisms (Lagassé et al., 2017). Moreover, proteins need to maintain their three-dimensional structure to be biologically active, which involves not only the correct sequence of amino acids but also proper folding and specific post-translational modifications (Chaudhary et al., 2017). Since therapeutic proteins are synthesized using cell-based production systems, complex purification strategies are generally involved. Several contaminants including DNA, cell debris, endotoxins, host cell proteins, media components, and viruses must be removed. Additionally, protein modification increases the complexity of purification even further, as more impurities may be added to the process, such as unreacted protein, reagents, or multiple less functional isomers. Currently, most purification processes for modified therapeutic proteins are based on chromatographic techniques. This may be explained by its multiple advantages over other methods, such as having a high resolution and robustness, and presenting high recovery levels (Przybycien et al., 2004; Gottschalk et al., 2012). To obtain the highest yield and purity, selection of a specific chromatographic technique and optimization of operating conditions are essential.

Numerous previous reviews describe the history, types of reactions, and purification strategies used for modified therapeutic proteins (Pfister and Morbidelli, 2014; Jafari et al., 2017; Jiang et al., 2018; Ramos-de-la-Peña and Aguilar, 2020; Duivelshof et al., 2021). However, they focus mainly on modified therapeutic proteins that have not reached the market yet and are still under development. This review presents a critical analysis of the chromatography-based purification strategies exclusively for all modified therapeutic proteins that have obtained regulatory approval and are currently commercialized. Based on such analysis, this review provides a guide for the selection of chromatography-based purification processes for novel modified therapeutic proteins. The information of the purification processes was mainly obtained from patents, as a primary source, or from the original reports associated to the design of the biopharmaceutical. This work is divided by modification type: PEGylation, Fc-fusion, and lipidation, while glycosylation and albumin-fusion are discussed in a single section given the low number of biopharmaceuticals from these categories currently on market. Within each section, a brief introduction of the modification strategy is presented, followed by an analysis of the purification process parameters (i.e., chromatographic technique, stationary phase, elution mode, etc.). Afterwards, coagulation factor IX, the only therapeutic protein with three different modifications on the market, is presented as a case study to demonstrate how the modification greatly influences the development of chromatographic processes. Finally, the current challenges to develop more efficient chromatography-based purification processes are summarized.

## PEGylation

PEGylation is the linking of one or more PEG molecules to a protein by means of a covalent bond (Pfister and Morbidelli, 2014). PEG is a synthetic polymer comprising repeating units of ethylene oxide with the following general molecular structure: HO-(CH_2_-CH_2_-O)_n_-H. It is a biocompatible, neutral, highly water-soluble, and flexible polymer that exhibits a random coil conformation (Fee and Van Alstine, 2011). After the PEGylation reaction, proteins acquire new features, such as increased physical and thermal stability, increased water solubility, proteolytic protection, and extended circulation half-life; properties that enhance the native protein’s pharmacokinetics and therapeutic efficacy (Pfister and Morbidelli, 2014).

**FIGURE 2:**
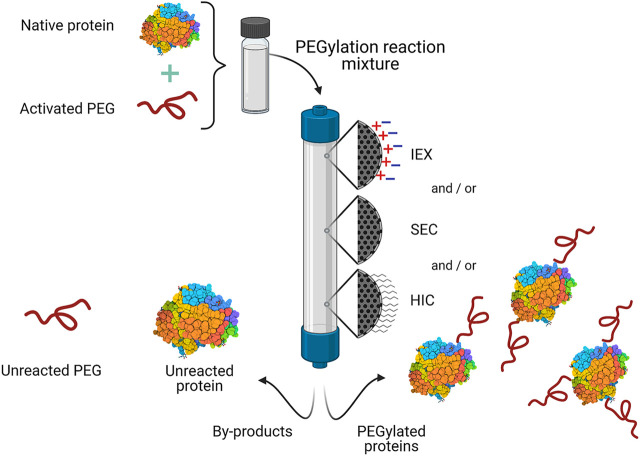
General scheme for the purification of commercial PEGylated proteins by chromatography. One or more chromatographic operational modes are used to separate the desired PEGylated protein(s) from the PEGylation reaction mixture. IEX, ion exchange chromatography; HIC, hydrophobic interaction chromatography; SEC, size exclusion chromatography.

PEGylation can be carried out using different strategies depending on the nature of the protein and desired application. The covalent attachment of PEG chains occurs at chemically reactive residues, often exposed at the surface of the protein, including lysine, cysteine, serine, histidine, arginine, tyrosine, threonine, aspartic acid, and glutamic acid; or at the N- and C-terminus. For this reaction to occur, the PEG chain must be functionalized at one end with an active group (activated PEG), which is chosen depending on the available residue(s) in the protein. It is worth mentioning that, prior to PEG conjugation, the protein must be already pure to increase the yield of the reaction (Pfister and Morbidelli, 2014; Ramos-de-la-Peña and Aguilar, 2020). Nevertheless, depending on the PEGylation reaction strategy, the products may include a heterogenous mixture of mono-, multi-PEGylated products (proteins with a varying number of attached PEG molecules), and/or positional isomers (proteins with the same number of PEG chains that differ from each other in the location of the PEG molecule). All of these differ in physicochemical and pharmaceutical properties (Swierczewska et al., 2015). Moreover, at the end of the PEGylation reaction unreacted PEG and native (unmodified) protein may still be present. PEGylated species and reactants are separated mainly by chromatography in its different operational modes (Figure 2).

PEGylation is a highly successful modification strategy as reflected by the large number of PEGylated proteins that have been approved and are available on the market. Table 1 summarizes the purification processes for these proteins, which largely depend on a specific protein and PEGylation reaction. It should be mentioned that in large-scale manufacturing processes, the purification strategy employed (chromatography mode or stationary phase) might be different to that reported at lab-scale/early development. Moreover, it is important to notice that not all the purification processes of PEGylated proteins rely on chromatographic techniques. In the cases of Adagen®, Revcovi™ and Palynziq®, only an ultrafiltration step is employed to remove unreacted PEG (Davis et al., 1981; Vellard et al., 2008; Filpula and Sapra, 2014; Bell et al., 2017). The conformation and size of these conjugates (PEG chains of 14, 13 and 36 kDa linked to Adagen®, Palynziq® and Revcovi™, respectively) allow their separation from the native protein and unreacted PEG by ultrafiltration membranes (Molek and Zydney, 2006). Unfortunately, for some PEGylated proteins, specifically biosimilar products of Neulasta®, the information regarding their purification processes is not available.

**TABLE 1:** Approved PEGylated proteins.

International non-proprietary name	Brand name	Protein	Treatment	Company	CT	Stationary phase	Approval year	References
Pegademase bovine	Adagen^®^	ADA	ADA severe combined immunodeficiency	Enzon Pharmaceuticals Inc.	—	—	1990	Davis et al. (1981)
Pegaspargase	Oncaspar^®^	L-asparaginase	Acute lymphoblastic leukemia	Enzon Pharmaceuticals Inc.	AEX	NA	1994	Turecek et al. (2016)
Peginterferon alfa-2b	ViraferonPEG^®^	IFN alfa-2b	Chronic hepatitis C	Schering-Plough	CEX	TSKgel SP-5PW	2000	Gilbert and Cho, (1998)
Peginterferon alfa-2a	Pegasys^®^	IFN alfa-2a	Chronic hepatitis B, C	Hoffman-La Roche	CEX	Toyopearl CM-650S, TSKgel SP-5PW	2001	Karasiewicz et al. (1995)
Peginterferon alfa-2b	PEG-intron^®^	IFN alfa-2b	Chronic hepatitis C	Schering-Plough	CEX	TSKgel SP-5PW	2001	Gilbert and Cho (1998)
Pegfilgrastim	Neulasta^®^	G-CSF	Neutropenia	Amgen	CEX	SP Sepharose HP	2002	Molineux (2004); Bailon (2008)
Pegvisomant	Somavert^®^	GH receptor antagonist	Acromegaly	Pfizer	HIC-CEX	Phenyl Toyopearl 650M, SP Sepharose FF	2003	Clark et al. (1996)
PEG-epoetin beta	Mircera^®^	Erythropoietin (epoetin-beta)	Anemia in adults with chronic renal failure	Hoffman-La Roche	CEX	SP Sepharose FF	2007	Burg et al. (2011)
Certolizumab pegol	Cimzia^®^	Anti-TNF-alfa Fab	Inflammatory diseases	UCB Pharma	CEX	SP Sepharose HP	2008	Chapman et al. (1999)
Pegloticase	Krystexxa^®^	Uricase	Chronic gout	Savient Pharmaceuticals	AEX	Mono Q	2010	Sherman et al. (2004); Williams et al. (2003)
Peginterferon alfa-2b	Sylatron™	IFN alfa-2b	Melanoma (post-surgical resection)	Merck	CEX	NA	2011	Park et al. (2019)
Lipegfilgrastim	Lonquex^®^	G-CSF	Neutropenia	Teva	NA	NA	2013	Awwad et al., 2018
Peginterferon beta-1a	Plegridy^®^	IFN beta-1a	Relapsing forms of multiple sclerosis	Biogen	SEC- CEX	Superose 6, SP Sepharose FF	2014	Pepinsky et al. (2001); Pepinsky et al. (2005)
PEG-growth hormone	Jintrolong^®^	Human growth hormone	Growth hormone deficiency	GeneScience	AEX	Q Sepharose	2014	Jin et al., 2012
Rurioctocog alfa pegol	Adynovate^®^	Coagulation factor VIII	Hemophilia A	Shire	SEC	Superose 6 HR	2016	Bossard et al. (2012)
Nonacog beta pegol	Rebinyn^®^	Coagulation factor IX	Hemophilia B	Novo Nordisk	AEX	POROS 50 HQ	2017	Wiendahl et al. (2020)
Calaspargase pegol	Asparlas™	L-asparaginase	Acute lymphoblastic leukemia	Servier Pharmaceuticals	NA	NA	2018	Marini et al., 2017
Elapegademase	Revcovi™	ADA	ADA severe combined immunodeficiency	Leadiant Biosciences	—	—	2018	Ramos-de-la-Peña and Aguilar, (2020)
Damoctocog alfa pegol	Jivi^®^	Coagulation factor VIII	Hemophilia A	Bayer	CEX	SP (Cytiva)	2018	Mei et al. (2010)
Pegvaliase	Palynziq^®^	Phenylalananine ammonia lyase	Phenylketonuria	BioMarin	—	—	2018	Park et al. (2019)
Rurioctocog alfa pegol	Adynovi^®^	Coagulation factor VIII	Hemophilia A	Baxalta Innovations	CEX	MacroCap SP	2018	Siekmann et al. (2011)
Pegfilgrastim jmdb	Fulphila™	G-CSF	Neutropenia	Mylan Pharmaceuticals	CEX	NA	2018	Hoy, (2019)
Pegfilgrastim cbqv	Udenyca™	G-CSF	Neutropenia	Coherus Bioscience	NA	NA	2018	Park et al. (2019)
Pegfilgrastim	Lapelga	G-CSF	Neutropenia	Apotex Inc.	NA	NA	2018	Zalipsky and Pasut, 2020
	Pelgraz™							
Pegfilgrastim	Pelmeg™	G-CSF	Neutropenia	Mundipharma	NA	NA	2018	Zalipsky and Pasut, 2020
Pegfilgrastim bmez	Ziextenzo™	G-CSF	Neutropenia	Sandoz Inc.	NA	NA	2019	Zalipsky and Pasut, 2020
Turoctocog alfa pegol	Esperoct^®^	Coagulation factor VIII	Hemophilia A	Novo Nordisk	AEX	Source 15Q	2019	Stennicke et al. (2013)
Ropeginterferon alfa-2b	Besremi	IFN alfa-2b	Polycythemia vera	PharmaEssentia	CEX	SP Sepharose XL	2019	Lin and Widmann, (2013)
Pegfilgastrim apgf	Nyvepria	G-CSF	Neutropenia	Pfizer	CEX	NA	2020	Yang et al. (2021)

ADA, adenosine deaminase; AEX, anion exchange chromatography; CEX, cation exchange chromatography; CT, chromatographic technique; FF, fast flow; G-CSF, granulocyte colony stimulating factor; GH, growth hormone; HIC, hydrophobic interaction chromatography; HP, high performance; HR, high resolution; IFN, interferon; NA, information not available; SEC, size exclusion chromatography; TNF, tumor necrosis factor. Notice that IFN alfa-2b is approved for different treatments according to dosage and presentation form.

### Chromatography-Based Approaches

The main chromatographic technique used to purify approved and commercialized PEGylated proteins is ion exchange chromatography (IEX), either anion exchange (AEX) or cation exchange (CEX) chromatography (Table 1). IEX has been extensively used in downstream processes for capture, intermediate and polishing steps (Pfister and Morbidelli, 2014). This chromatographic technique allows the simultaneous separation of the native protein, unreacted PEG, mono- and multi-PEGylated species, and even positional isomers (Seely and Richey, 2001; Gašperšič et al., 2016; Nguyen et al., 2016). The PEGylation reaction can alter the superficial charge of the protein. The PEG chain produces a “charge shielding effect” and generates weak electrostatic interactions with the resin (Nguyen et al., 2016). Furthermore, if PEGylation takes place on a charged amino acid residue within the protein (i.e., lysine or arginine), this is neutralized and in consequence the isoelectric point (pI) of the conjugated protein is altered. Moreover, hydrogens from the PEG chain can form hydrogen bonds with some functional groups of the protein and raise the pKa (Fee and Van Alstine, 2011).

High resolution and purity can be obtained using IEX by choosing optimal operating conditions. These conditions are the operation mode (bind-elute or flow-through), elution mode (linear or step gradient), stationary phase, sample load, mobile phase (type, concentration, and pH), elution type (ionic or pH), elution buffer (type, concentration, and pH), gradient length, flow rate, and column dimensions (Ahamed et al., 2008). From the information available on purification processes for commercial PEGylated proteins, the operation mode and elution methods are highlighted. Most of commercial PEGylated proteins are purified using a linear gradient elution, with a bind-elute operation mode. A common method for protein elution in IEX is using a salt gradient. In IEX, protein adsorption is driven by electrostatic interactions between the stationary phase and the proteins (Shi et al., 2005). These interactions are in turn affected by the nature and concentration of salt (ionic strength) in the mobile phase. As the salt concentration increases, the retention of the protein decreases as a consequence of loss of electrostatic interactions (Staahlberg et al., 1991). Salt gradients generated by increasing sodium chloride (NaCl) concentration (in a range from 0 to 1 M) prevail the most in the purification processes of commercial proteins (Neulasta®, Somavert®, Krystexxa®, Mircera®, Cimzia®, Plegridy®, Adynovi®, and Esperoct®) (Table 1). Only for the case of PEG-intron® (a 12 kDa-PEG interferon alfa-2b for chronic hepatitis C), the ionic strength of the mobile phase was modified by increasing the buffer components concentration (from 10 to 80 mM Na_3_PO_4_). The salt concentration range used to elute a PEGylated protein must be carefully selected. It will depend on the surface net charge of the protein and how it is affected by the modification. When the PEG chain slightly alters the surface net charge of the protein, it is desirable to use a narrow concentration range of NaCl. This can be seen in the purification process of PEG-epoetin beta (Mircera®), where the column was equilibrated with 100 mM of NaCl and the PEGylated protein was eluted with 200 mM NaCl (Burg et al., 2011).

An alternative method to salt gradient for protein elution in IEX is to use a pH gradient. pH gradients are required to separate proteins with small differences in the isoelectric point (pI), which can be generated by positional isomers of PEGylated species. Cation exchange resins featuring weak acid groups have been used effectively to purify proteins exploiting pH gradients (Pabst and Carta, 2007; Pabst et al., 2008). For instance, in the downstream processing of Pegasys® (a 40 kDa-PEG interferon alfa-2a for chronic hepatitis B and C), it was possible to separate five different positional isomers by using a Toyopearl CM-650S resin (column dimensions: 16 mm diameter and 120 cm length) at a flow rate of 21 cm/h and eluting with a linear pH gradient (from 4.0 to 7.8). Additionally, a preparative column packed with an analytical matrix (TSKgel SP-5PW) was used to increase the purity of the isoforms above 88% (Foser et al., 2003). In the purification process of Rebinyn® (a 40 kDa-PEG coagulation factor IX for hemophilia B), a pH gradient is generated by equilibrating the column with acetate buffer 100 mM pH 5.7, followed by a five column volumes (CV) acidic wash with acetate buffer 250 mM pH 4.3; then elution is accomplished by a 5 CV linear gradient of 10 mM histidine, 50 mM NaCl, 50 mM CaCI_2_ buffer pH ≈ 6.0 (Stennicke et al., 2008; Wiendahl et al., 2020). Nevertheless, this approach, based on the affinity between the different buffer components to the stationary phase, is poorly implemented due to its drawbacks in practical applications. Because the column re-equilibration process becomes more complicated since the counterion of the elution buffer has a higher affinity to the support than the initial counterion (present in the equilibrium buffer), it is necessary to flush the column with the initial buffer at a higher concentration, and subsequently re-equilibrate the resin to the buffer’s initial concentration (Staby et al., 2009; Weiss, 2019).

On the other hand, size exclusion chromatography (SEC) is another chromatographic technique well recognized for the purification of PEGylated proteins. The PEGylation process of a protein significantly alters its molecular weight and size. A simple addition of the PEG molecular weight and the protein molecular weight will give a reasonable approximation of the final molecular weight of the conjugate. However, this is not the case for the hydrodynamic radius. A single PEG molecule, when attached to a protein of the same molecular weight, will increase the hydrodynamic radius of the resulting conjugate by more than double (Fee and Van Alstine, 2004). The elution pattern of PEGylated proteins in SEC is related to molecular size rather than molecular weight (Fee, 2009). This chromatographic technique is particularly useful for the removal of low molecular weight species from the reaction mixture (i.e., excess reagents, functionalized PEG hydrolysis products or PEGylation by-products) and separation of unreacted PEG molecules from the native protein (Jevševar et al., 2010; Fee and Van Alstine, 2011). However, it is less effective for the separation of multi-PEGylated species. SEC can effectively separate two PEGylated species with *n* and *n*-1 PEG chains, but only up to *n* = 3. For multi-PEGylated species (*n* > 3), its ability to resolve PEG conjugates differing by a single PEG chain is practically null (Fee and Van Alstine, 2006; Fee and Van Alstine, 2011). Moreover, the resolving power of SEC is notoriously greater when the protein is attached to high molecular weight PEG chains (Sherman et al., 1997).

To obtain an efficient separation between the native protein and its PEGylated counterpart by SEC, a general rule of thumb is that they must have a molecular weight difference of at least two-fold (Fee, 2009). For Plegridy® (a 20 kDa-PEG interferon beta-1a indicated for multiple sclerosis), the purification process after PEGylation consists of two sequential chromatography steps: SEC followed by CEX. In the SEC step, the PEGylation reaction mixture is loaded into a Superose 6 resin and results in the separation of the mono-PEGylated conjugate from the native protein (Pepinsky et al., 2001; Pepinsky et al., 2005). The nominal molecular weight of the PEG reactant (20 kDa) and the molecular weight of interferon beta-1a (∼23 kDa) are basically equal, resulting in a conjugate with doubled molecular weight compared to that of the native protein. The downstream process of Adynovate® (a 20 kDa-di-PEGylated coagulation factor VIII for hemophilia A) consists of a single SEC step, in which Superose 6 is used as a stationary phase (Bossard et al., 2012).

Hydrophobic interaction chromatography (HIC) is also used to purify PEGylated proteins. The hydrophobicity of a protein is altered by attachment of PEG chains, either increasing or decreasing it, depending on the native’s protein hydrophobicity (Fee and Van Alstine, 2006). Studies have shown that the change in hydrophobicity between the native and PEGylated proteins is sufficient to separate these species (Cisneros-Ruiz et al., 2009). However, the hydrophobicity difference that allows an efficient separation by HIC among mono- and multi-PEGylated species (including positional isomers) is only large enough when the attached PEG has a high molecular weight (>20 kDa) (Mayolo-Deloisa et al., 2012; Huang et al., 2013). Hence, HIC may not exhibit a clearly defined elution profile of mono- and multi-PEGylated species or positional isomers when small PEG molecular weight is used (Pfister and Morbidelli, 2014), holding back the use of HIC in large-scale purification of PEGylated proteins in comparison to IEX or SEC. Only in the downstream processing of Somavert® (a 5 kDa-multi-PEGylated growth hormone receptor antagonist for acromegaly), HIC is used to remove small amounts of high molecular weight cross-linked and unreacted products. In this process, a Phenyl Toyopearl column is equilibrated with 0.35 M sodium citrate, 0.05 M Tris pH 7.6, and the PEGylated protein is eluted with a six CV linear gradient from 0.35 to 0.0 M sodium citrate. Afterwards, a desalting step using G-25 Sephadex is carried out and finally, CEX is used to separate the multi-PEGylated species (Clark et al., 1996; Olson et al., 1997). Usually, the equilibration phase in HIC is carried out at high salt concentration to expose the hydrophobic nuclei, although the optimal salt concentration to achieve protein binding will depend upon the hydrophobicity of the resin and target protein. The growth hormone receptor antagonist presents a high degree of hydrophobicity and the ligand of the resin (phenyl group) is also highly hydrophobic, therefore, mild salt conditions are required to bind the protein to the resin. Unfortunately, for many proteins mild salt conditions are not enough for binding and other salts, like ammonium sulphate, most be used at higher concentrations. This reason also accounts to the drawback for HIC implementation at large scale.

### Stationary Phases

The selection of a chromatography media for large-scale purification of a protein is not trivial. There are different aspects to consider about the stationary phase, such as physicochemical properties and commercial aspects (costs and supply chain management) (Cooney, 1984). Features such as backbone matrix, ligand, particle size distribution, and pore size will determine the physical and chemical properties of the chromatographic media. To achieve a good performance in the purification of PEGylated proteins, the chromatographic resin should meet several aspects such as high hydrophilicity, good chemical and physical stability, large pore size according to the target PEGylated protein, high binding capacity and resolution, narrow particle size distribution, and avoid non-specific interactions (Huang et al., 2013).

The purification of commercial PEGylated proteins is carried out mainly using agarose-based resins, followed by polymethacrylate-based resins (Table 2). The mechanical strength of the resin is largely dependent on the material of the backbone matrix, particle size distribution, and particle porosity (Nweke et al., 2017). Agarose and methacrylate-based matrices are often employed in ion exchangers for industrial scale purification of therapeutic proteins (Pabst et al., 2007; Liu et al., 2010). Agarose is commonly used as a backbone material because its manufacture and customization (i.e., porosity and functionalization) is relatively straightforward (Nweke et al., 2017). Additionally, the high degree of hydroxylation of this natural polymer makes it highly hydrophilic, which prevents non-specific interactions with proteins (Jungbauer, 2005). Typically, during its manufacturing agarose is cross-linked to build a more rigid structure with enhanced mechanical stability (Jungbauer, 2005; Nweke et al., 2017). Conversely, cellulose-based matrices have a macro-porous structure which can offer high binding capacities. However, this kind of matrix is not used for the purification of commercial PEGylated proteins. This may be due to the fact that in comparison to agarose-based resins, cellulose-based matrices are hard to pack, and present deficient flow performance (Levison et al., 1996; Jungbauer, 2005). In large-scale production, good flow performance is crucial since it allows to achieve shorter processing times.

**TABLE 2:** Structural properties of resins used to purify commercial PEGylated proteins. Information is based on manufacturer’s data sheets unless cited otherwise.

Stationary phase	CT	Ligand	Backbone matrix	Particle size (µm)	Pore size (nm)	Manufacturer
Q Sepharose HP	Strong AEX	Quaternary ammonium (Q)	6% CLA	24–44	70^a^	Cytiva
Source 15Q			Polystyrene/divinyl benzene	15	20–1000^b^	Cytiva
Mono Q			Polystyrene/divinyl benzene	10	100^c^	Cytiva
POROS 50 HQ		Quaternized polyethyleneimine	Polystyrene/divinyl benzene	50	1726^d^	Thermo Scientific™
TSKgel SP-5PW	Strong CEX	Sulfopropyl (SP)	Polymethacrylate	10	100	Tosoh
SP Sepharose HP			6% CLA	24–44	70^a^	Cytiva
SP Sepharose FF			6% CLA	45–165	60^a^	Cytiva
SP Sepharose XL			CLA, with dextran surface extender	45–165	12^e^	Cytiva
MacroCap SP			Acrylamide-dextran copolymer	50	100^f^	Cytiva
Toyopearl CM-650S	Weak	Carboxymethyl (CM)	Polymethacrylate	35	100	Tosoh
	CEX					
Phenyl Toyopearl 650M	HIC	Phenyl	Polymethacrylate	65	100	Tosoh
Superose 6	SEC	—	Highly CLA	30–40	25–29^g^	Cytiva

AEX, anion exchange chromatography; CEX, cation exchange chromatography; CLA, cross-linked agarose; CT, chromatographic technique; FF, Fast Flow; HIC, hydrophobic interaction chromatography; HP, high performance; NA, information not available; SEC, size exclusion chromatography.

a Avallin et al., 2016

b Miyabe and Guiochon, 2000

c Mant and Hodges, 1991

d Yao and Lenhoff, 2006

e Yao and Lenhoff, 2004

f Huang et al., 2013

g Nweke et al., 2017.

On the other hand, resins based on synthetic polymers, such as polymethacrylate and polystyrene/divinylbenzene have also been used for PEGylated proteins purification (Table 2). They present several advantages over natural polymer-based resins, including resistance to extreme chemical conditions (pH and oxidizing environments), greater mechanical strength, and larger pore sizes (Carta and Jungbauer, 2010). Acrylamide-dextran copolymer used in MacroCap SP resin, is another matrix worth mentioning. MacroCap SP was designed especially for the purification of PEGylated molecules at large-scale, with operating conditions at 120 cm/h, with an optimum bed height of 15–30 cm (Fee and Van Alstine, 2011; Huang et al., 2013).

### Particle and Pore Size

The particle size of the resin, depending on the type of operation, impacts the chromatographic resolution and the dynamic binding capacity, and influences the pressure drop (Carta and Jungbauer, 2010). A wide range of particle sizes (10–165 μm) and pore sizes (12–1726 nm) are used to purify commercial PEGylated proteins (Table 2). Notice that resins with small particle sizes (∼30 μm) have a large pore size (all of them correspond to synthetic-based matrices), while resins with large particle sizes (∼90 μm) have a small pore size (all of them correspond to agarose-based matrices). Large particles (>50 μm) provide low pressure drops and ease the packing and manufacturing process compared to small particles (Jungbauer, 2005). However, when large beads and high flow rates are used, the chromatographic efficiency depends almost completely on mass transfer kinetics (Carta et al., 2005). On the contrary, small particles (20–50 μm) are usually employed for large-scale purifications that require a high efficiency (Carta and Jungbauer, 2010). Beads with a narrow particle size distribution (PSD) may result in less backpressure and higher resolution (Huang et al., 2013). This is the case for resins such as Source 15Q, Mono Q and POROS 50 HQ, which were designed for fast high-resolution ion exchange protein separations.

The pore size of the resin plays an important role in mass transfer kinetics, which may be negatively affected if the pore size is either close or smaller than the target protein. To obtain fast, high-resolution separation processes, high mass transfer rates are required. This may be achieved with beads featuring highly interconnected, large pores. However, large pores may also reduce surface area, and subsequently decrease resin capacity (Jungbauer and Hahn, 2008). Since PEGylated proteins have a large molecular size, the selection of an appropriate resin pore size is of great importance. To overcome the hindered diffusion effect exerted by PEG chains in conventional resins with pore sizes <100 nm, macroporous resins have emerged as a technological solution. These resins can be operated at high flow rates without compromising efficiency and capacity (Qu et al., 2008). MacroCap SP, a macroporous resin, is used in the downstream processing of Adynovi® (indicated for hemophilia A) (Siekmann et al., 2011). By using MacroCap SP, 100 nm pore size, it is possible to purify the full-length coagulation factor VIII (280 kDa) linked to two 20 kDa PEG chains (Turecek et al., 2012). Resins with a similar pore size that have been used to purify other commercial PEGylated proteins with similar or smaller molecular weights include, Mono Q, TSKgel SP-5PW, Toyopearl CM-650S and Phenyl Toyopearl 650M (see Tables 1, 2).

Perfusion chromatography media has also proven to be effective for the purification of PEGylated proteins. This type of resin overcomes problems associated with mass transfer in the separation of large molecules. It is constituted by two sets of pores: through-pores and diffusive pores, promoting a better access to the inside of the particle by the combination of convective and diffusive flow (García et al., 2000). POROS 50 HQ, a perfusion resin, is used in the downstream processing of 98 kDa Rebinyn® (Wiendahl et al., 2020). The large pore size of POROS 50 HQ (1726 nm) enhances mass transfer rates due to intraparticle convection and diffusion without compromising the binding capacity (Yao and Lenhoff, 2006). Matlschweiger et al. demonstrated that the binding capacity of thyroglobulin (680 kDa) is five times larger when using POROS 50 HQ rather than Q Sepharose FF (60 nm pore size), evidencing the promising balance between transport kinetics and binding capacity of perfusion resins (Matlschweiger et al., 2019).

Sepharose XL is an agarose-based media grafted with dextran to increase exposure and density of ion-exchangers ligands, resulting in higher loading capacities than those of Sepharose FF materials (Yao and Lenhoff, 2006). As a consequence of the dextran chains filling the pores, SP Sepharose XL has a pore size of 12 nm, five times smaller than its non-grafted analogue SP Sepharose FF. Despite its small pore size, this resin has shown to be effective for separating PEGylated proteins. This may be attributed to the presence of dextran chains, which make the adsorption sites to be flexible, enhancing the binding capacity and allowing a better resolution (Hubbuch et al., 2003; Zhu and Carta, 2014; Angelo and Lenhoff, 2016). This resin is used in the downstream processing of Besremi®, an interferon alfa-2b linked to a 40 kDa-branched PEG indicated for the treatment of polycythemia vera (Lin and Widmann, 2013).

### Trends in Chromatographic Supports for PEGylated Proteins Purification

Currently, there is a great number of chromatography media on the market; however, there is not a single resin that completely meets all the optimum requirements to achieve a high resolution separation. Continuous advancements in materials, polymer science, and ligand chemistry are boosting the development of more robust and versatile resins to achieve a good separation and high resolution under a broad range of experimental conditions. For instance, Zhai et al. developed gigaporous polystyrene/divinylbenzene-based agarose-grafted particles (Agap-co-PS) with a narrow PSD (30–85 μm) and vast network of interconnected through-pores and diffusive pores (100–500 nm). By using a sulfopropyl-modified version of these particles as chromatography media, the purification of PEGylated G-CSF (granulocyte colony stimulating factor) was improved in terms of purity and yield compared to MacroCap SP resin (Qu et al., 2008; Zhai et al., 2012). Another interesting alternative for the purification of PEGylated proteins is to use monolithic chromatographic supports. They possess highly connected open pores allowing convective mass transfer and high flow rates. The ability of monoliths to separate PEGylated proteins by IEX and HIC modes has been demonstrated in previous reports (Gašperšič et al., 2016; Mayolo-Deloisa et al., 2016).

The modification of chromatography media with novel ligands has also been investigated as a strategy to improve PEGylated proteins purification. Different chromatographic supports have been grafted with PEG molecules of varying molecular weights to improve resolution and even separate positional isomers of mono-PEGylated species by exploiting the called “hydrophobicity-shielding effect” (Hernández-Martínez and Aguilar, 2014; Sánchez-Trasviña et al., 2019). Heparin has also been proposed as a novel ligand to separate PEGylated proteins by affinity chromatography. Mejía-Manzano et al. used Heparin Sepharose 6 FF to purify PEGylated lysozyme through a NaCl step gradient reaching a 100% of yield and purity (Mejía-Manzano et al., 2017).

Process integration is a trend in biopharmaceutical production used to diminish costs and manufacturing times. In this context, on-column PEGylation has emerged as a strategy to merge the PEGylation reaction, separation, and purification in a single step. Furthermore, on-column PEGylation may result in a more homogeneous mixture of PEG conjugates. This methodology can be applied practically with all chromatography modes, but it has been extensively explored with IEX in recent years (Maiser et al., 2015; Pfister et al., 2016; Wang et al., 2017). The next step forward is to switch from a batch-mode on-column PEGylation to a continuous process. Ingold et al. demonstrated that by implementing a reactive multicolumn countercurrent solvent gradient purification (rMCSGP) process, it was possible to obtain mono-PEGylated alpha-lactalbumin with a higher yield (85%) and productivity compared to batch on-column PEGylation (Ingold et al., 2016).

## Fc-Fusion

Fc-fusion proteins are composed of an immunoglobulin Fc domain that is genetically fused to a protein of interest, such as a protein domain, enzyme, ligand or peptide (Yang et al., 2018). If a single protein molecule is bonded to the Fc domain (which consist of a dimeric form of CH2-CH3 domain), the Fc-fusion protein is said to be monomeric, whereas dimeric Fc-fusion proteins consist of two protein molecules fused to the Fc domain, resembling the structure of natural immunoglobulins (Rath et al., 2015). The Fc domain confers new biological and pharmacological properties to the protein, such as extended circulation half-life, enhanced solubility and stability, and modulated immunogenicity, and enhanced Fc-mediated effector functions (Czajkowsky et al., 2012; Yang et al., 2018). Although there are several types of immunoglobulins, IgG is the most used for constructing Fc-fusion proteins, probably because it has the longest circulation half-life (Jazayeri and Carroll, 2008). At present, twelve Fc-fusion proteins have obtained regulatory approval and are available on the market (Table 3).

**TABLE 3:** Approved Fc-fusion proteins.

International non-proprietary name	Brand name	Protein	Treatment	Company	CTS	Stationary phase	Approval year	References
Etanercept	Enbrel^®^	p75 TNF receptor (ED)	Rheumatoid arthritis, autoimmune and inflammatory diseases	Boehringer/Pfizer	AF-IEX-NA	NA	1998	Hassett et al. (2018); Mohler et al. (1993)
Abatacept	Orencia^®^	CTLA-4 (ED)	Rheumatoid arthritis	Lonza/Bristol-Myers	AF-NA-NA-NA	NA	2005	Linsley et al. (1998)
Rilonacept	Arcalyst^®^	IL-1R accessory protein	Cryopyrin-associated periodic syndromes	Regeneron Pharmaceuticals	AF-SEC	NA	2008	Economides et al. (2003)
Romiplostim	NPlate^®^	Thrombopoietin receptor agonist	Thrombocytopenia in chronic immune thrombocytopenic purpura patients	Amgen/Pfizer	CEX- CEX	SP Sepharose FF-SP Sepharose HP	2008	Liu et al. (2017)
Aflibercept	Eylea^®^	VEGF receptors 1 and 2 (BD)	Wet age-related macular degeneration, diabetic macular edema	Regeneron Pharmaceuticals	AF-CEX- AEX-HIC	NA	2011	Assadourian et al. (2014)
Belatacept	Nulojix^®^	CTLA-4 (ED)	Organ rejection	Bristol-Myers	AF-AEX-HIC	MabSelect Protein A, Q Sepharose FF, Toyopearl Phenyl 650M	2011	Leister et al. (2010)
Alefacept	Amevive^®^	LFA-3 (ED)	Psoriasis and organ rejection	Astellas Pharma	AF-SEC	Protein A-Sepharose 4B, Superose 12	2011	Miller et al. (1993); Wallner et al. (1999)
Ziv-aflibercept	Zaltrap^®^	VEGF receptors 1 and 2 (BD)	Metastatic colorectal cancer	Regeneron Pharmaceuticals	AF-CEX-AEX-HIC	NA	2012	Assadourian et al. (2014)
Efmoroctocog alfa	Eloctate^®^	Coagulation factor VIII (B-domain deleted)	Hemophilia A	Biogen	AF-AEX- HIC	VIIISelect, Fractogel EMD TMAE Hicap, Octyl Sepharose 4 FF	2014	Dumont et al. (2012); McCue et al. (2015)
Eftrenonacog alfa	Alprolix^®^	Coagulation factor IX	Hemophilia B	Biogen	AF-AEX- AEX	MabSelect SuRe, Fractogel DEAE, Q Sepharose	2014	McCue et al. (2014); Peters et al. (2010)
Dulaglutide	Trulicity^®^	GLP-1 receptor agonist	Glycemic control in type 2 diabetes mellitus	Eli Lilly and Co.	AF-SEC	Protein A Sepharose HP, Superdex 200	2014	Glaesner et al. (2010)
Luspatercept-aamt	Reblozyl^®^	Activin receptor Type IIB (Modified ED)	Anemia in adult with beta thalassemia	Celgene Corp/Acceleron Pharma Inc.	AF-AEX	MabSelect SuRe, Q-Sepharose	2019	Sako et al. (2010)

AEX, anion exchange chromatography; AF, affinity chromatography; BD, binding domain; CEX, cation exchange chromatography; CTLA-4, cytotoxic lymphocyte-associated antigen 4; CTS, chromatographic techniques sequence; ED, extracellular domain; GLP-1, glucagon-like peptide-1; HIC, hydrophobic interaction chromatography; IL-1, interleukin-1; LFA-3, leukocyte function antigen-3; NA, information not available; SEC, size exclusion chromatography; TNF, tumor necrosis factor; VEGF, vascular endothelial growth factor.

Fc-fusion proteins are produced by genetic engineering using a wide variety of expression systems. Mammalian systems are often preferred because they maintain proper folding of the protein, are able to perform post-translational modifications, and they allow easy extracellular secretion of the protein, which simplifies the downstream process (Jazayeri and Carroll, 2008). Among commercial Fc-fusion proteins, only NPlate® (a thrombopoietin receptor agonist to treat thrombocytopenia) is produced in a prokaryotic system. In addition to the impurities related to the recombinant production of Fc-fusion proteins, such as host cell proteins (HCP), virus, and DNA, several other contaminants are generated (Shukla and Gottschalk, 2013); for instance, one problem often encountered with Fc-fusion proteins is the formation of high molecular weight aggregates (HMWA) (Vaniotis et al., 2018). Also, low molecular weight species (LMWs), which include an incomplete Fc domain, may be produced (Zhu et al., 2019). To ensure safety and efficacy of the final product, it is critical to have a robust downstream process that maximizes the removal of process and product-related impurities.

**FIGURE 3:**
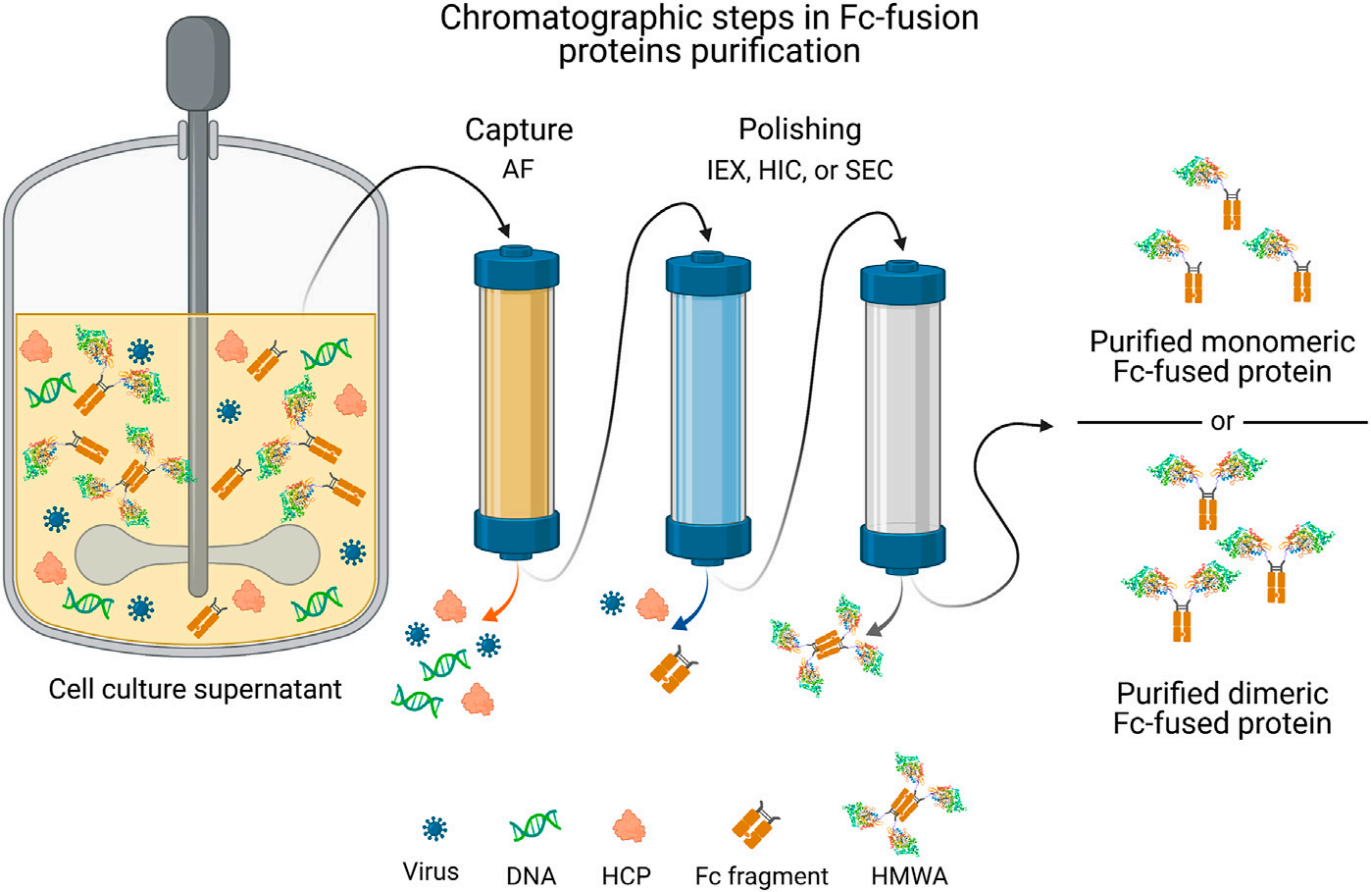
General strategy of chromatographic steps in the purification process of commercial Fc-fusion proteins produced in mammalian cells culture. A single capture step is followed by two polishing steps that involve a combination of chromatographic operational modes to obtain the purified monomeric or dimeric Fc-fusion protein. AF, affinity chromatography; HCP, host cell proteins; HIC, hydrophobic interaction chromatography; HMWA, high molecular weight aggregates; IEX, ion exchange chromatography; SEC, size exclusion chromatography. Fc-fusion protein structures are merely representative, the current structure for each Fc-fusion protein may vary.

Given the structural resemblance between Fc-fusion proteins and monoclonal antibodies (mAbs), the purification approaches employed for the latter may serve as a base platform to purify Fc-fusion proteins. However, biochemical properties (i.e., pH-sensitivity, pI, and aggregation-propensity) between these molecules substantially differ, requiring specific adjustments in the downstream process for Fc-fusion proteins (Shukla and Norman, 2017). The general scheme of the purification process for mammalian system-expressed Fc-fusion proteins (Figure 3) involves: i) a capture step, which aims to eliminate most HCP, DNA, and other cell culture-related impurities; ii) an intermediate step for viral inactivation; and iii) a polishing step, oriented to remove any remaining impurities and HMWA (Shukla and Thömmes, 2010). It is important to highlight that the structure of Fc-fusion proteins may vary (Duivelshof et al., 2021). The downstream processing for Fc-fusion proteins typically includes three chromatographic steps, often an affinity chromatography for capture, and two different chromatographic techniques (IEX, HIC or SEC) for polishing.

### Capture Step

Most commercial Fc-fusion proteins use protein A affinity chromatography as an initial capture step in their downstream processing, except for Nplate® and Eloctate® (a B domain-deleted coagulation factor VIII for hemophilia A) (Table 3). This technique relies on the highly specific interaction between the Fc domain of antibodies with protein A ligand. A single step of protein A chromatography may result in >98% purity even with complex cell culture media as a starting material (Shukla et al., 2007). Protein A chromatography is effective in removing process-related contaminants such as HCP, DNA, and virus, relieving the burden on subsequent intermediate and polishing steps (Pinto et al., 2015).

Regardless of its advantages, protein A chromatography faces some issues related to pH sensitivity of Fc-fusion proteins. Generally, the protein binds to the column at neutral pH, and elutes at acidic pH (Ghose et al., 2006; Jazayeri and Carroll, 2008). Low pH values may negatively impact the integrity and biological activity of Fc-fusion proteins, or promote the formation of HMWA (Shukla et al., 2007). Product aggregation may be prevented by addition of stabilizers, such as arginine or glycine into the elution buffer (Arakawa et al., 2004). Whereas the use of chaotropic salts, such as urea, or guanidine hydrochloride may be utilized as “disaggregation” agents to dissociate HMWA and recover functional monomers (Xu et al., 2010).

Several other limitations can also be pointed out regarding protein A chromatography, including resin cost, throughput, and resin instability (Fahrner et al., 1999; Follman and Fahrner, 2004). A wide variety of Protein A resins are commercially available. However, costs between $8,000–15,000 USD per liter for this type of resins translate into higher expenses compared to traditional resins using non-protein-based ligands (Ramos-de-la-Peña et al., 2019). Furthermore, due to the high cost of the resin, typical bioprocesses rely on running several cycles of short-column Protein A chromatography steps to purify a single batch of product, increasing total purification time and decreasing production rate (Ghose et al., 2007). This results in multiple cleaning, sanitization, and resin regeneration cycles, involving harsh acidic and alkaline conditions, which can modify Protein A three-dimensional structure and/or lead to ligand leaching (Jiang et al., 2009). The acidic environment during elution also shortens resin’s lifespan. To overcome these drawbacks manufacturers have continuously engaged in the design of novel resins with improved characteristics. MabSelect SuRe™, used in the capture step of Reblozyl® (activin receptor type IIB to treat beta thalassemia) and Alprolix® (a coagulation factor IX for hemophilia B), is a resin functionalized with a tetrameric chains of alkali-stabilized protein A-derived ligand (of the Z-domain of staphycoccal protein A) produced in *E. coli* (Silva et al., 2018). This ligand is NaOH tolerant, allowing a deep cleaning of the resin after each purification round, minimizing cross-contamination, and extending the lifetime of the resin. Hahn et al. found that Protein A leaching in MabSelect SuRe™ remained at low levels (<3 ppm) over 50 purification and cleaning (using 500 mM NaOH) cycles, while product recovery and purity remained constant (Hahn et al., 2006). Moreover, by optimizing the cleaning and resin regeneration protocols, the lifetime of MabSelect SuRe™ could be prolonged up to 150 cycles, which may translate into economical savings in large-scale production (Zhou et al., 2019). Recently, new resin with protein A ligand designed by protein engineering (MabSelect^TM^ PrismA) showed greater stability for regeneration process (Wetterhall et al., 2021), however, this chromatographic material has not been reported to purify Fc-fusion proteins.

Currently, a great number of commercial protein A resins with differences in the nature of the ligand (native or recombinant), valency (which dictates the Fc-fusion protein-protein A stoichiometry) and features (backbone structure, particle, and pore size distribution) are available. Most of the resins employed in the capture step of commercial Fc-fusion proteins possess similar structural properties: same backbone structure (cross-linked agarose), similar particle size (∼85 μm) and pore size (∼120 nm) (Table 4). These features provide high surface area, large sample load, and low back pressure which are required during the capture step. Highly cross-linked agarose media can manage high flow rates, making it suitable for large-scale applications (Hahn et al., 2003; Ghose et al., 2007). Moreover, large pore sizes allow a better diffusion of modified proteins that have a molecular weight in the range between 72 and 220 kDa. Among the protein A resins utilized in the capture step of commercial Fc-fusion proteins, only one has a small pore and particle size: Protein A Sepharose HP, used for Trulicity®. Trulicity® is a glucagon-like peptide-1 (GLP-1) receptor agonist used for glycemic control in type 2 diabetes mellitus that has a molecular weight of 63 kDa. The pore size of Protein A Sepharose HP (70 nm) is large enough to enable protein diffusion through the pores. Additionally, the small particle size (34 μm) of this resin enhances peak resolution, facilitating subsequent purification steps.

**TABLE 4:** Structural properties of resins used to purify commercial Fc-fusion proteins. Information is based on manufacturer’s data sheets unless cited otherwise.

Stationary phase	CT	Ligand	Backbone matrix	Particle size (µm)	Pore size (nm)	Manufacturer
Q Sepharose HP	Strong AEX	Quaternary ammonium (Q)	6% CLA	24–44	70^a^	Cytiva
Q Sepharose FF			6% CLA	45–165	60^a^	Cytiva
Fractogel EMD TMAE HiCap		Trimethylammoniumethyl (TMAE)	Polymethacrylate	40–90	80	MerckMillipore
Fractogel EMD DEAE	Weak AEX	Diethylaminoethyl (DEAE)	Polymethacrylate	40–90	80	MerckMillipore
SP Sepharose HP	Strong CEX	Sulfopropyl (SP)	6% CLA	24–44	70^a^	Cytiva
SP Sepharose FF				45–165	60^a^	Cytiva
Phenyl Toyopearl 650M	HIC	Phenyl	Polymethacrylate	65	100	Tosoh
Octyl Sepharose 4 FF		Octyl	4% CLA	45–165	110^a^	Cytiva
MabSelect Protein A	AF	Recombinant protein A (*E. coli*)	Highly CLA	40–130	120^a^	Cytiva
Protein A Sepharose HP		Protein A	6% CLA	24–44	70^a^	Cytiva
MabSelect SuRe		Alkali-stabilized Protein A-derived (*E. coli*)	Highly CLA	85	120^a^	Cytiva
Protein A Sepharose 4B		Native protein A	4% CLA	45–165	110^a^	Cytiva
VIII Select		13 kDa recombinant protein (*S.cerevisae*)	Highly CLA	75	NA	Cytiva
Superose 12	SEC	—	Highly CLA	30–40	10–14^b^	Cytiva
Superdex 200		—	CLA and dextran	34	13^b^	Cytiva

AEX, anion exchange chromatography; CEX, cation exchange chromatography; CLA, cross-linked agarose; CT, chromatographic technique; FF, fast Flow; HIC, hydrophobic interaction chromatography; HP, high performance; NA, information not available; SEC, size exclusion chromatography.

a Avallin et al., 2016

b Hagel et al., 1996.

Besides protein A, other ligands may be utilized for the recovery of Fc-fusion proteins. Particularly, in the capture step of the downstream processing of Eloctate® (a beta-domain deleted coagulation factor VIII), VIIISelect resin is employed. The ligand of this resin is a *Camelidae*-derived 13 kDa recombinant nanobody produced in *Saccharomyces cerevisiae*, which binds specifically to beta-domain-deleted factor VIII molecules (Dumont et al., 2012; McCue et al., 2015). The use of VIIISelect allows the product to be eluted at neutral pH conditions, which may help maintain protein integrity (McCue et al., 2015).

An exception is observed in the downstream process of Nplate®, since it does not include a capture step *per se*. Nplate® is expressed as inclusion bodies in *E. coli*, where proteins can reach up to 90% purity. Prior to chromatography steps, inclusion bodies are recovered, solubilized, and incubated in an appropriate buffer to promote protein refolding and dimerization of the monomer subunits through the correct formation of disulfide bridges (Alibolandi and Mirzahoseini, 2011; Liu et al., 2017). After clarification of the refolding reaction mixture, polishing chromatography steps are employed to remove misfolded proteins, along with HMWA, and HCP (Shukla and Norman, 2017).

### Polishing Step

Following capture of Fc-fusion proteins, one or two additional chromatography steps are usually employed as polishing steps. While protein A chromatography is a straightforward choice for the capture step of Fc-fusion proteins, the selection of the chromatographic techniques and sequence of polishing steps directly depends on the remaining product and impurities to be removed (Pinto et al., 2015). Polishing steps are used to remove misfolded proteins, remaining HCP, DNA, LMW, HMWA, viral particles, and leached Protein A (Shukla et al., 2007; Shukla and Norman, 2017). In addition, to achieve enough viral inactivation, an acid hold step is introduced post protein A chromatography (Shukla and Aranha, 2015). However, the duration of this step must be carefully determined, as Fc-fusion products may suffer detrimental effects if held in low pH environments for too long. After viral inactivation, the product pool must be neutralized before the next polishing step. In some occasions, this pH adjustment step may aid to remove some HCP by precipitation, however it can also sacrifice product yield (Li, 2017). To avoid some of the risks associated with a low-pH environment, solvents or detergents may also be used for viral inactivation (Conley et al., 2017).

For most commercial Fc-fusion proteins, IEX predominates as a first polishing step (Table 3). This inexpensive and rapid chromatographic technique allows to remove HCP, DNA, viral particles, leached protein A, and charge variants (Liu et al., 2010). Since most Fc-fusion proteins have a pI less than 7, AEX in a bind-elute mode is a great choice for removing impurities (Shukla and Gottschalk, 2013). The product pool is first loaded onto the anion exchange column and the protein is then eluted by a salt or pH gradient. Acidic impurities, such as DNA, HCP, and viral particles also bind to the resin, and are eluted during cleaning or regeneration steps (Liu et al., 2010). AEX has also been employed in a weak partitioning mode. In this operation mode, pH and ionic strength are selected to enhance binding of the protein and impurities to the resin. Since impurities are more acidic than the protein they bind tightly to the resin, which leads to an increase in impurity removing performance (Liu et al., 2010; Li, 2017). AEX operated in a weak partitioning mode has proven to improve HCP and HMWA removal (Shukla and Gottschalk, 2013; Shukla et al., 2017). CEX can also be applied for the purification of Fc-fusion proteins, specifically for those with pI values ranging from neutral to basic. This technique is typically operated in a bind-elute mode. In contrast to AEX, acidic impurities do not bind to the resin since they are negatively charged and thus, are removed in the load and wash fraction (Liu et al., 2010).

After the first polishing step, a second or third step may be incorporated depending on remaining impurities and product characteristics. AEX in flow-through mode may be used if HCP and HMWA have reached satisfactory levels and the remaining impurity is DNA (Shukla and Gottschalk, 2013). Another alternative for subsequent polishing steps is using HIC. This chromatographic technique is incorporated as the last polishing step of the purification processes of Eloctate®, Zaltrap® (a vascular endothelial growth factor for metastatic colorectal cancer), Eylea® (a vascular endothelial growth factor to treat age-related macular degeneration), and Nulojix® (a cytotoxic lymphocyte-associated antigen 4 for organ rejection). HIC is often used to remove HMWA since it exploits hydrophobic interactions between aggregates and the resin (Shukla et al., 2017; Shukla and Norman, 2017). It is typically operated in a flow-through mode due to low binding capacity of HIC resins (Shukla and Gottschalk, 2013). Bind-elute protocols have also proven to be effective. For instance, HIC is used in a bind-elute mode in the downstream processing of Nulojix®. The eluate from the previous AEX step is loaded onto a HIC column in a 50 mM HEPES buffer pH 7.0 and 3.6 M ammonium sulfate (AS). Then, weakly bound impurities are removed through a washing step using a concentration of 1.2 M AS. Finally, the target Fc-fusion protein is eluted at 0.55 M, while HMWA remain bound to the resin (Leister et al., 2010).

Lastly, SEC is also used as a polishing step, although to a lesser extent than AEX or CEX. This chromatographic technique is employed in the downstream processing of Arcalyst® (an Interleukin-1 accessory protein for cryopyrin-associated periodic syndromes), Amevive® (a leukocyte function antigen-3 for psoriasis and organ rejection) and Trulicity® (Table 3). SEC allows to effectively separate HMWA, Fc fragments and reduce HCP from the desired product, and at the same time helps to desalt the Fc-fusion protein solution. Due to the low load capacity of SEC columns, this chromatographic technique is typically used as the final stage of the purification process (Baumann and Hubbuch, 2017; Schmitt et al., 2017). To obtain high purity levels, a high chromatographic resolution is required, which may be achieved by using resins with small and uniform particle size (Cytiva, 2010). From Table 4, it is remarkable that SEC resins used in the downstream processes of commercial Fc-fusion proteins possess small particle size distributions (30–40 μm) and small pore sizes (10–14 nm).

An interesting polishing scheme is observed for Alprolix®, which involves two sequential AEX steps, instead of two orthogonally different chromatographic techniques. The second AEX step is operated in a pseudo-affinity mode with the objective of recovering recombinant coagulation factor IX (Peters et al., 2010; McCue et al., 2014). Pseudo-affinity chromatography originated based on the observations that calcium ions could induce conformational changes in some proteins, modifying their hydrophobicity and/or surface charge. These changes affect the normal interaction of a protein towards conventional IEX or HIC supports (Yan, 1996). Fischer et al. observed that implementing an AEX step followed by an AEX run in pseudo-affinity mode allowed a 4-fold increase in recombinant coagulation factor IX specific activity compared to a single AEX polishing step (Fischer et al., 1995).

### Trends in Fc-Fusion Purification Strategies

Since downstream processing represents the bottleneck of biopharmaceuticals manufacturing in general, there is a continuous interest in improving the performance of chromatographic resins. In recent years, mixed-mode chromatography (MMC) has gained attention as a chromatographic technique for polishing steps and even as a substitute of protein A affinity chromatography in capture steps. Mixed-mode resins are unique stationary phases as their ligands are capable of simultaneously establishing different types of interactions with biomolecules, i.e., electrostatic, hydrophobic, and to some extent hydrogen bonding (Chu et al., 2018). MMC has been effective for clearance of HCP, DNA, leached protein A, and even HMWA. When the target Fc-fusion protein is highly sensitive to low pH values to use protein A chromatography, MMC can be used as a capture step instead, since protein elution is accomplished at less acidic conditions (pH ∼4.0) (Shukla and Gottschalk, 2013). Several commercial MMC resins have been explored as protein A chromatography alternatives for the capture of Fc-fusion proteins, including MEP HyperCel, HEA HyperCel, PPA HyperCel, Capto MMC and Capto adhere. These resins have shown good product recovery and HCP clearance (Pezzini et al., 2011; Toueille et al., 2011; Joucla et al., 2013; Shukla and Gottschalk, 2013; Kaleas et al., 2014).

The implementation of MMC may also lead to the simplification of downstream processing of Fc-fusion proteins by reducing the number of chromatographic steps, which results in cost savings and improvement of process efficiency (Rathore et al., 2018). By using MMC as the sole polishing step instead of CEX followed by AEX; Herzer et al. were able to simplify the purification process of a domain antibody fragment-Fc fusion, achieving the required HCP clearance (<100 ppm) and purity (Herzer et al., 2015). In this context, a single MMC step was able to replace two IEX polishing steps.

Ceramic hydroxyapatite (CHT) is an archetype of MMC that combines cation exchange and metal affinity. It has positively charged calcium and negatively charged phosphate groups that bind proteins via carboxyl clusters and cationic exchange (Premsukh et al., 2011). In the downstream processing of antibodies, CHT has demonstrated a superior ability to reduce antibody aggregates and HCP (Li, 2017). When CHT is used in polishing steps, relatively mild elution conditions, such as phosphate gradients (10–500 mM) at neutral pH are required (Gagnon, 2009).

Lastly, increasing throughput in downstream processing of biopharmaceuticals is an urgent need in the industry. The implementation of membrane chromatography in Fc-fusion proteins manufacturing has become a feasible option to address this issue. Membrane adsorbers feature larger pore sizes than chromatographic resins. Hence, mass transfer faces less resistance as convection predominates over solute diffusion, allowing membranes to be operated at high flow rates under high loading conditions, and shortening the processing cycle (Shukla and Norman, 2017). However, as a consequence of large pore size there is a decrease in surface area, which translates into poor protein binding capacities compared to packed-bed chromatography (Gagnon, 2012). Due to this limitation, membrane adsorbers are often employed in flow-through mode. IEX-based membrane chromatography has been explored as an intermediate polishing step in mAb and Fc-fusion proteins production, as it contributes to clearance of large impurities such as host DNA and viral particles (Liu et al., 2013; Orr et al., 2013). Moreover, when IEX based membrane is applied as a pre-filtration method, it may improve parvovirus filtration (Brown et al., 2010). Precisely this strategy is followed in the downstream processing of Eloctate®, where Mustang Q (an AEX-membrane adsorber) is employed after an AEX chromatography step and prior to virus filtration. In addition to its high throughput, membrane chromatography also possesses an advantage from an economical perspective. Membrane adsorbers are inexpensive and usually commercialized in single-use, disposable formats, which may diminish costs and time required for validation and routine implementation of cleaning and regeneration protocols of multi-use chromatography resins (Hou et al., 2015).

## Lipidation

Lipidation, a natural process that takes place in eukaryotic cells, is a post translational modification in which a lipid moiety is covalently attached to a protein. Among the lipid molecules used for protein modification are long-chain fatty acyl groups, isoprenoids, sterols, phospholipids, lipid-derived electrophiles, and glycosylphosphatidylinositol, which are conjugated to proteins in cells by specific transferases (Resh, 2013; Chen et al., 2018). Typically, conjugation to fatty acyl groups (acylation) or isoprenoids occurs at nucleophilic side chains (mainly cysteine, serine and lysine residues) or the N-terminus of proteins, while modification with cholesterol or glycosylphosphatidylinositol occurs at the C-terminus (Jiang et al., 2018).

Lipidation of therapeutic proteins is mainly carried out through N-acylation with long-chain fatty acids, as a strategy to improve the biomolecule stability and circulation half-life (Longo et al., 2014). Half-life extension is given by the non-covalent interaction between the fatty acid moiety and human serum albumin (HSA), one of the most abundant proteins in plasma, which has a half-life of several weeks (Knudsen and Lau, 2019). Once the fatty acid moiety of the lipidated protein binds to HSA, the lipidated protein is protected from proteolytic degradation and renal clearance (Dalbøge et al., 2015). Furthermore, lipidated proteins have shown to self-assemble into oligomeric complexes, especially in the presence of zinc ions. These complexes are less susceptible to proteolytic degradation, and work as a prolonged release mechanism, since drug monomers gradually dissociate from the complex (Havelund et al., 2004; Jonassen et al., 2012; van Witteloostuijn et al., 2016).

Lipidation, as a half-life extension strategy, has been primarily implemented for small proteins and peptides. During the modification process, the hydrophobicity of the native protein is increased by the attachment of a fatty acid chain. This effect is greater in small proteins and peptides in comparison to larger proteins (Frokjaer and Otzen, 2005). Additionally, lipidation is recognized as a safe modification, since the fatty acids used for this purpose (i.e., myristic and palmitic acids) can be completely metabolized by the human body (van Witteloostuijn et al., 2016). To date, five lipidated therapeutic proteins are available on the market (see Table 5) for the treatment of type 1 and type 2 diabetes mellitus and related conditions (i.e., obesity and glycemic control). They are composed of either insulin or GLP-1 analogues conjugated to a single long-chain fatty acid. All these therapeutic proteins are manufactured by Novo Nordisk, one of the pioneers in protein lipidation (Kurtzhals et al., 1995).

**TABLE 5:** Approved proteins with other modifications: albumin-fusion, glycosylation, and lipidation.

Modification	International non-proprietary name	Brand name	Protein	Treatment	Company	CTS	Stationary phase	Approval year	References
Albumin-fusion	Albutrepenonacog alfa	Idelvion^®^	Coagulation factor IX	Hemophilia B	CSL Behring	AEX-AF-MMC	Q Sepharose FF- Heparin support-CHT	2016	Metzner et al. (2009)
Glycosylation	Darbepoetin alfa	Aranesp^®^	Erythropoetin (analogue)	Anemia due to chronic kidney disease or myelosuppresive chemotherapy	Amgen Inc.	AEX- RPC, SEC	Q Sepharose FF, Vydac C4, Bio-Sil SEC 250	2001	Elliot and Byrne, (1997)
Lipidation	Insulin determin	Levemir^®^	Insulin (analogue)	Type 1 and 2 diabetes mellitus	Novo Nordisk	RPC	NA	2005	Havelund et al. (1997)
	Liraglutide	Victoza^®^	GLP-1 (analogue)	Type 2 diabetes mellitus and obesity	Novo Nordisk	CEX	Source 30S	2009	Staby, (2011)
	Liraglutide	Saxenda^®^	GLP-1 (analogue)	Management of obesity	Novo Nordisk	CEX	Source 30S	2010	Staby, (2011)
	Insulin degludec	Tresiba^®^	Insulin (analogue)	Type 1 and 2 diabetes mellitus	Novo Nordisk	AEX, RPC	Source 15Q, Nucleosil C4	2013	Jonassen et al. (2009)
	Semaglutide	Ozempic^®^	GLP-1 (analogue)	Type 2 diabetes mellitus and obesity	Novo Nordisk	RPC	C18	2017	Lau et al. (2012)
	Somapacitan-beco	Sogroya^®^	Human growth hormone (analogue)	Growth hormone deficiency	Novo Nordisk	NA	NA	2020	Thygesen et al. (2017)

AEX, anion exchange chromatography; AF, affinity chromatography; CEX, cation exchange chromatography; CHT, ceramic hydroxyapatite; CTS, chromatographic techniques sequence; FF, fast flow; GLP-1, glucagon-like peptide-1; NA, information not available; SEC, size exclusion chromatography; RPC, reverse phase chromatography.

**FIGURE 4:**
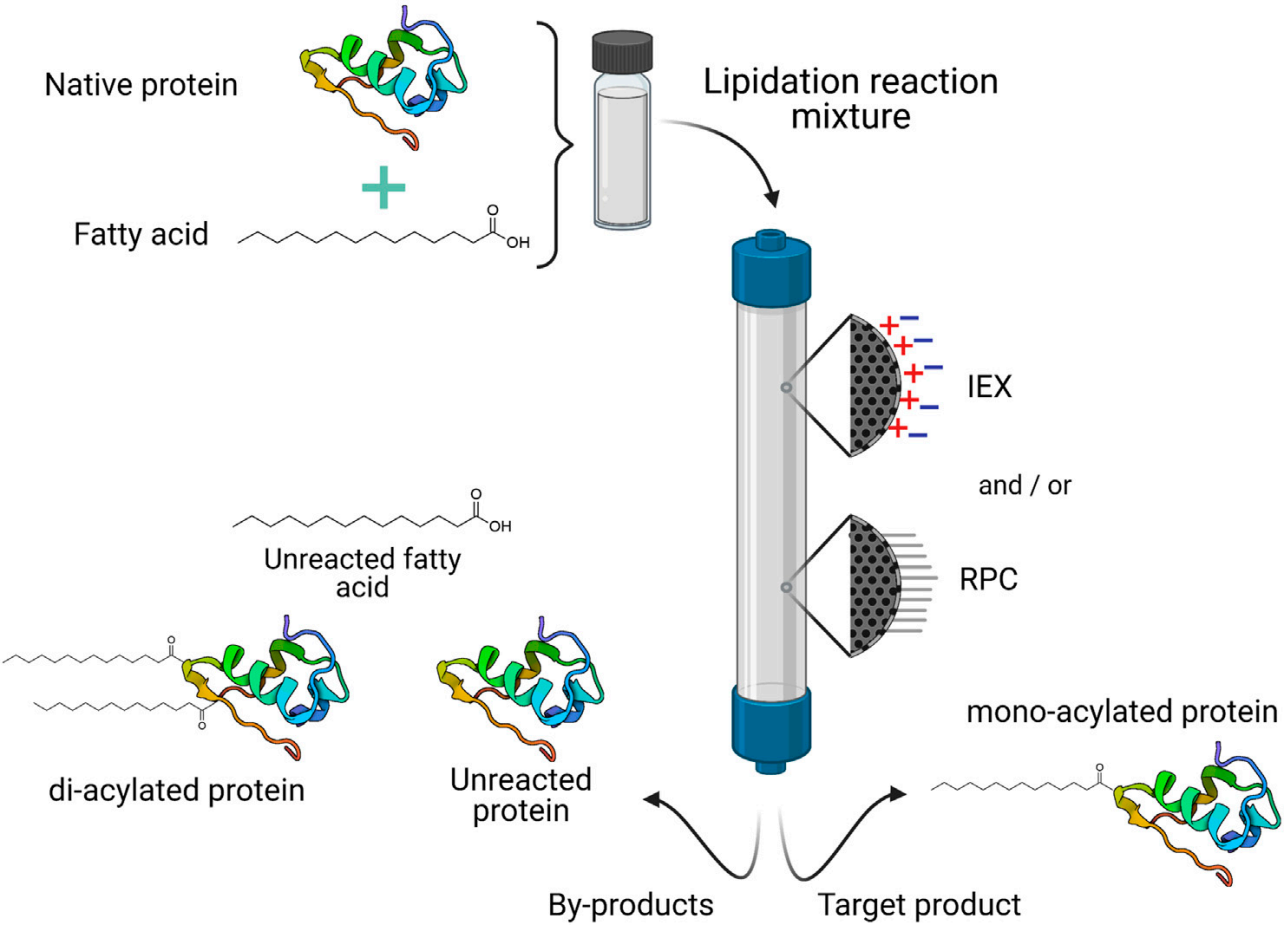
General purification process for commercial lipidated proteins. One or two different chromatographic operational modes may be necessary to isolate the target product (usually the mono-acylated protein) from the lipidation reaction mixture. IEX, ionic exchange chromatography; RPC, reverse phase chromatography.

Similar to PEGylation, lipid conjugation is achieved through chemical reactions between a reactive fatty acid derivative and a pure therapeutic protein. The lipid moiety may be directly attached to an amino acid residue, or via a spacer such as L-glutamic acid. Due to the hydrophobic nature of lipid reactants, in some cases it may be necessary to carry out the reaction in polar organic solvents, or in a mixture of aqueous and water-miscible organic solvents. Solvent selection will depend on the properties and stability of the protein or peptide, and the solubility of reagents (Havelund et al., 1997; Knudsen et al., 2001; Jonassen et al., 2009). The lipidation reaction strategy will influence the chromatographic strategies followed to purify lipidated proteins. Among the main contaminants to be removed after the conjugation reaction are the unmodified protein, di-acylated forms, and unreacted fatty acid (Figure 4).

### Purification Strategies

Reverse phase chromatography (RPC) is employed in most of the purification processes of marketed lipidated proteins, whether as a single purification step (as in the cases of Levemir® and Ozempic®) or in combination with IEX (Table 5). RPC employs solid supports, usually silica particles, modified with alkyl chains (mainly C_4_, C_8_ or C_18_) to render a hydrophobic stationary phase, which can strongly interact with hydrophobic or low-polarity molecules (Schlüter, 1999; Poole and Lenca, 2017). Since lipidation increases protein hydrophobicity, RPC is particularly useful for the separation of lipidated derivatives from the unmodified protein. In this type of chromatography, elution is carried out by a mixture of water and water-miscible organic solvents with different polarity. Lipidated proteins are eluted by a concentration gradient of ethanol or acetonitrile (Havelund et al., 1997; Schlüter, 1999; Jonassen et al., 2009; Lau et al., 2012). As the solvent concentration increases, the polarity of the mobile phase decreases, and the partition of lipidated proteins will be favored towards the mobile phase and it will elute (Schlüter, 1999).

Among the modified therapeutic proteins discussed in this review, lipidated proteins are the smallest conjugates in terms of molecular weight. Tresiba® (an hexadecandioyl-conjugated insulin analogue for the treatment of type 1 and type 2 diabetes mellitus), the largest of this class, has a molecular weight of 6.1 kDa. Additionally, the impurities after the acylation reaction are mainly low molecular weight species as opposed to the high molecular weight contaminants in PEGylation (i.e., unreacted PEG and multi-PEGylated species) or HMWA found in Fc-fusion proteins. The small size of the target conjugates allows the use of stationary phases featuring low particle sizes (≤30 μm) for RPC and IEX, which contribute to separations with high resolution. In all the cases where RPC is used for the purification of lipidated proteins, semi-preparative HPLC columns are employed. Semi-preparative RP-HPLC is an excellent technique for the purification of small proteins and peptides offering a high resolution (purification factor between 2 and 200) even at large scales (Schlüter, 1999; Josic and Kovac, 2010). Semi-preparative RP-HPLC columns feature low particle sizes (≤15 μm) with small pores (100–300 Å), which promote enhanced chromatographic resolution and column efficiency in comparison with other preparative chromatography techniques, such as IEX, SEC and HIC. Moreover, the silica-based matrix of RP-HPLC columns confers great mechanical stability, allowing operation at high flow rates (200–800 cm/h) at acceptable back-pressure values (Josic and Kovac, 2010).

IEX is also used in the purification processes of marketed lipidated proteins (Table 5). The use of this chromatographic technique follows two different approaches. On one hand, CEX is used as a single purification step for both Victoza® and Saxenda® (a palmitoyl-conjugated GLP-1 analogue), which are used for the treatment of type 2 diabetes mellitus and obesity management, respectively. On the other hand, AEX is used prior to RPC in the purification of Tresiba® (Jonassen et al., 2009; Staby, 2011).

The purification of Tresiba® begins with the precipitation of the crude product from the reaction mixture. Then, it is resuspended in an aqueous/ethanol buffer at pH 7.5 and loaded into a column packed with Source 15Q (Cytiva), a strong AEX resin (Jonassen et al., 2009). The pI of insulin degludec (the component in Tresiba®) is similar to that of native insulin, which is around 5.6 (Kohn et al., 2007; Wang et al., 2012). Therefore, at loading pH insulin degludec binds to the resin, while certain impurities are lost in the flow-through. Elution is performed with an ammonium acetate gradient, from 0.24 to 1.25% w/w, and target-containing fractions are subjected to purification by RP-HPLC using a Nucleosil C4 250/10 mm, 10 μm, 300 Å column, where elution is carried out by an ethanol gradient (from 7 to 42% v/v). The purity of insulin degludec after AEX is 72.9% and it increases to 99.4% after semi-preparative RP-HPLC. However, during the RP-HPLC step, 50% of the loaded target product is lost. Although semi-preparative RP-HPLC excels in delivering the target product at a superior purity, it does so at expenses of productivity. Therefore, a thorough optimization of this chromatographic technique is crucial to increase yield without compromising purity. Semi-preparative RP-HPLC combined with IEX is considered an essential method for the separation and characterization of proteins in a complex mixture (Josic and Kovac, 2010).

Staby described the use of CEX as a single chromatography step for the purification of both Victoza® and Saxenda® (Staby, 2011). The crude reaction mixture after acylation, comprising mono-, di-acylated and unmodified GLP-1, was loaded into a column packed with Source 30S (Cytiva), a strong CEX resin, equilibrated with an aqueous/ethanol buffer at pH 3.5. Using a linear salt gradient from 0 to 1.30% w/w KCl, the successful separation of di-acylated, mono-acylated (desired product) and unmodified GLP-1 (following this elution order) was achieved. This indicates that the acylation process modifies the protein’s physicochemical properties, specifically its surface net charge, making the use of IEX feasible for the purification of the target product.

An interesting aspect regarding the elution mode in IEX steps of Tresiba® and Victoza®/Saxenda®, is the narrow salt gradient used to elute proteins. The equilibrium and elution buffer in both cases contained a large concentration of ethanol: 42% w/w and 64.5% w/w for Tresiba® and Victoza®/Saxenda®, respectively. The addition of organic solvents to the mobile phase in IEX changes the selectivity and capacity factor by modifying the electrostatic interactions between the analytes and the stationary phase, contributing to the separation of analytes (Craig, 2012).

### Trends in Lipidated Proteins Purification

Regarding the chromatography strategies implemented for the purification of lipidated proteins, semi-preparative RP-HPLC is still the most prevalent. However, the application of a single IEX step, as in the case of Victoza® and Saxenda®, represents significant advantages, from which cost and time savings are the most relevant, given the lower costs and higher throughput of IEX compared to semi-preparative RP-HPLC (Knudsen et al., 2001).

Lipidation has proven to be successful, as evidenced by currently available commercial formulations. Although only acylated insulin and GLP-1 analogues have reached the market, the principle of lipidation has been applied and evaluated for other proteins and peptides, such as interferon alpha (Yuan et al., 2008) and desmopressin (Wang et al., 1999).

## Other Modifications

### Glycosylation

Glycosylation involves the covalent attachment of a carbohydrate moiety to a protein (Solá and Griebenow, 2010). It is one of the most important and complex post-translational modifications. Approximately 50–60% of all human proteins are glycosylated, either by the addition of N- or O-linked glycans (Roucka et al., 2018). Glycosylation has been used as an effective strategy to optimize therapeutic proteins efficacy (Solá and Griebenow, 2010). This is due to the fact that glycosylation has many functions including stabilization of protein conformation, control of protein folding, modulation of immunogenicity, and regulation of signal transduction, cell adhesion and half-life (Berger et al., 2011; van Witteloostuijn et al., 2016).

In nature, glycoproteins are obtained by a complex process involving several enzymes from different pathways (Berger et al., 2011). In counterpart, many glycosylated therapeutic proteins are produced by genetic engineering approaches using a wide variety of expression hosts that somehow mimic the human glycosylation pattern (van Witteloostuijn et al., 2016). However, proteins are often obtained as a mixture of multiple glycoforms, which may have significant variations in biological activity (Lingg et al., 2012; Roucka et al., 2018). To address this issue, several strategies have emerged, including enzymatic and chemical synthesis, and manipulation of biosynthetic pathways in different expression hosts (Li and Wang, 2018). Furthermore, glycosylation profiles are highly influenced by upstream and downstream processes conditions, making necessary systematic monitoring throughout development and manufacturing processes (Zhang et al., 2016).

Currently, there are several glycosylated therapeutic proteins available on the market. Most of them are either naturally glycosylated or their glycosylation pattern has been modified. In this review, focus will be placed in the purification process of marketed glycosylated therapeutic proteins containing additional glycosylation sites (added by genetic engineering approaches) to those occurring naturally. Until now, there is only one therapeutic protein available on the market that has been hyperglycosylated to optimize its biological activity: Aranesp®, which is an analogue of erythropoietin used for the treatment of anemia due to chronic kidney disease or myelosuppressive chemotherapy (Table 5). Aranesp® has a higher sialic acid content than native erythropoietin. Sialic acid is a negatively charged monosaccharide. In consequence, an increase of sialic acid in the protein can alter its surface charge and pI, which have been related to increased circulatory half-life of glycosylated proteins (Solá and Griebenow, 2010).

Aranesp® is produced using recombinant Chinese hamster ovary (CHO) cells. Thus, its downstream processing focuses on isolating the glycoform of interest, containing five N-linked oligosaccharide chains, and removing contaminants, such as DNA, cell debris, endotoxins, HCP, media components and viruses. First, cell culture media is loaded to an AEX column, packed with Q Sepharose FF resin (Cytiva), using an equilibration buffer (Tris 10 mM pH 8.5) that allows binding of the multiple erythropoietin glycoforms to the resin. The rationale behind the use of this technique is that the negative charge of the protein increases depending on its sialic acid content. The elution of glycoforms that remain bound to the AEX column is achieved by a 50 CV linear gradient of NaCl ranging from 0 to 0.5 M. A long linear gradient allows a better separation of protein glycoforms which are just slightly different. Afterwards, RPC is used as a first polishing step. The additional sialic acids in the protein alter its hydrophobicity, making the use of this technique feasible for the separation of glycoforms (Vreeker and Wuhrer, 2017). The fractions from AEX are pooled and loaded into a C4 column (Vydac®, Hichrom Limited). The elution of the glycoform of interest is accomplished by a 30 CV gradient of ethanol ranging from 20 to 94% v/v.

The last polishing step of Aranesp® involves the use of HPLC-SEC. Glycan moieties often contribute in a considerable manner to the mass of glycosylated proteins. Two different pools were obtained from the RPC step: one containing the glycoform of interest and another containing the glycoform of interest and aggregates. These pools were loaded separately to a HPLC-SEC column (Biosil SEC 250, Bio-Rad) and peaks corresponding to the monomeric glycoform were collected. The use of SEC for separation of glycosylated proteins and peptides presents special difficulties because their molecular size and shape are subjected to large variations with the mobile phase ionic strength and pH. The pH and ionic strength of the mobile phase should also be selected carefully to avoid interactions of the glycosylated proteins among each other or with the matrix of the stationary phase, which may result in distorted peaks and altered elution of the proteins (Churms, 2002). Although SEC may be performed at a large scale, its use is preferred in the final stages of protein purification. This is because at final purification stages, the sample volume is reduced making feasible the use of smaller SEC columns. Also, the use of smaller columns reduces run times and the amount of required chromatographic media, diminishing operating costs.

### Albumin-Fusion

Human albumin, produced mainly in the liver, is a highly water-soluble and the most abundant protein in plasma, with a very long circulation half-life (19 days on average) (Kratz, 2008). Due to these features, it has been used to improve the pharmacokinetic and physicochemical characteristics of some therapeutics. Proteins can be linked to albumin either by non-covalent interactions (by specific moieties that bind to albumin) or covalent bonds (by chemical conjugation or genetic engineering fusion) (Sleep et al., 2013). The attachment of proteins to albumin has been intensively studied in the last decades. However, until now, only one albumin-fusion therapeutic protein is available on the market: Idelvion® (coagulation factor IX to treat hemophilia B, Table 5).

The production of Idelvion® is carried out by extracellular expression in CHO cells. Thus, its purification process is focused on the clearance of HCP, nucleic acids, and other contaminants, following a very similar scheme to that used for Fc-fusion proteins. As a capture step, Q sepharose FF resin (Cytiva) is employed, and elution is accomplished by a NaCl/CaCl_2_ step gradient. The rationale of the use of Q Sepharose FF (Cytiva), a strong AEX support, and the elution with NaCl/CaCl_2_ in the capture step of Idelvion® is deeply discussed further in *Case Study: Purification of Modified Coagulation Factor IX*. A heparin affinity support is used as the first polishing step, using a NaCl step gradient elution. Heparin is a highly sulfonated (negatively charged) polydisperse linear polysaccharide. Chromatographic supports featuring immobilized heparin can be employed to purify different types of proteins, such as enzymes, serine protease inhibitors, growth factors, antithrombin III antibodies, extracellular matrix proteins, coagulation factors, DNA-binding proteins, lipoproteins, and hormone receptors (Xiong et al., 2008). Conveniently, heparin can act as an affinity ligand with biological specificity, but also as a cation exchanger (Xiong et al., 2008; Bolten et al., 2018).

The second and last polishing step of the purification process of Idelvion® is carried out using ceramic hydroxyapatite particles (CHT) (Bio-Rad) and a step gradient elution with sodium phosphate (Metzner et al., 2009). CHT particles are produced by spray-drying hydroxyapatite nanocrystals to obtain porous beads of 20–80 μm, which are suitable for protein chromatography. The beads are sintered at high temperature to stabilize the structure and gain mechanical stability. Depending on the sintering temperature, properties such as porosity and pore size may vary. A wide range of CHT resins are commercially available with different properties and applications (Wang and Carta, 2019). The type of CHT used in Idelvion® purification process is not reported; however, Bio-Rad commercializes two different types of CHT resins: type 1 and type 2. Both have a nominal particle size of 40 μm but differ in its porosity and surface area. CHT type 1 has a smaller pore size (30 vs. 49 nm) but a larger surface area (48 vs. 30 m^2^/ml particle) in comparison to CHT type 2 (Wang and Carta, 2019). CHT type 2 has been used to recover native coagulation factor IX and its recombinant form fused with prothrombin propeptide by showing high affinity and selectivity to the CHT support (Blostein et al., 2008).

## Case Study: Purification of Modified Coagulation Factor IX

**FIGURE 5:**
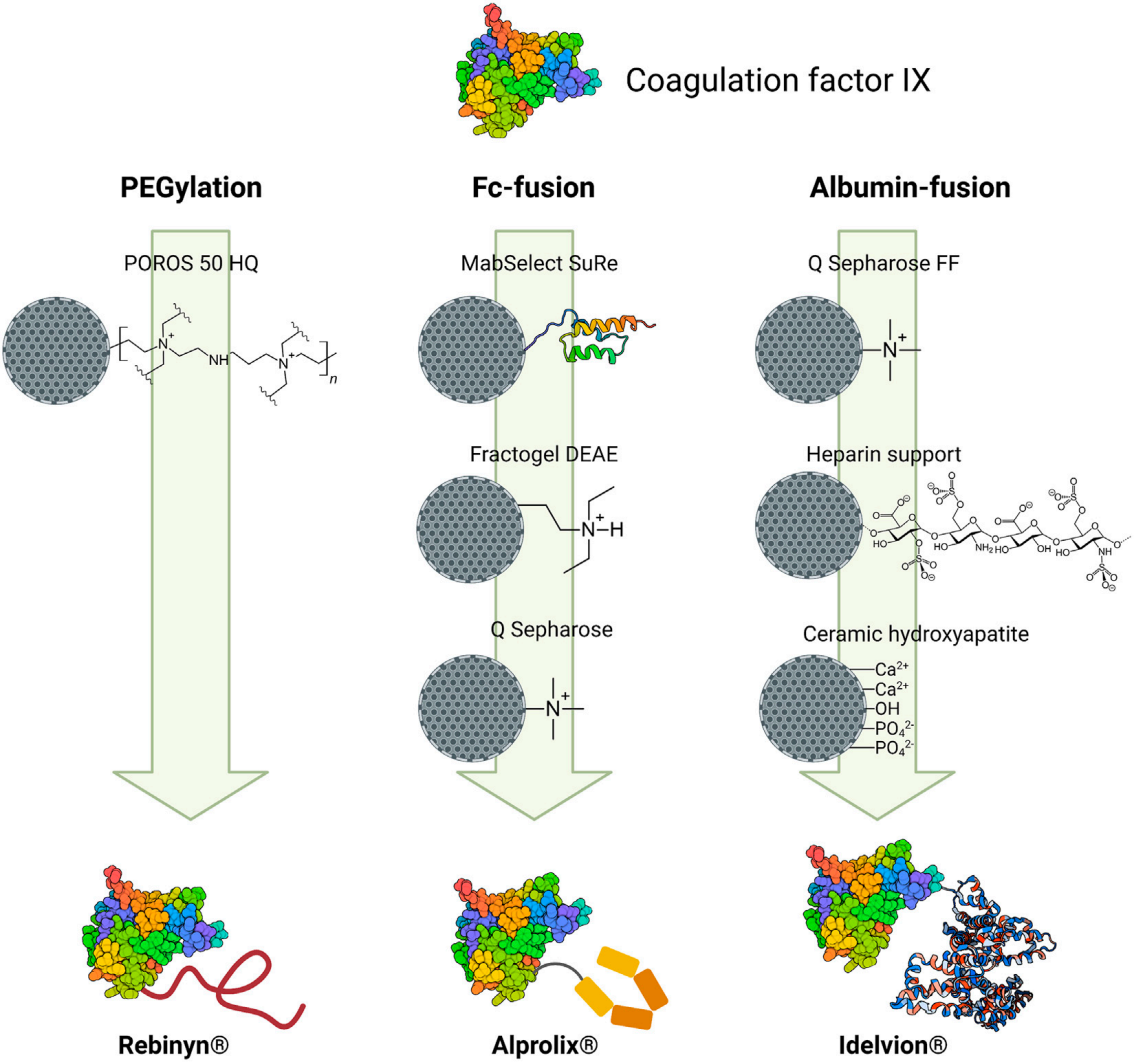
Chromatographic strategies followed to purify the three different versions of modified coagulation factor IX. For each modification, resins (depicting their ligands) are placed in order according to the downstream process. The modified proteins representations are illustrative, and they do not reflect the actual location of the conjugation.

Coagulation factor IX (FIX), a protein with a molecular mass around 57 kDa, plays a key role in the blood coagulation system. The deficiency of FIX produces hemophilia B, which causes frequent spontaneous bleeding episodes in different tissues (Metzner et al., 2009; Santagostino et al., 2012). To treat hemophilia B, different FIX products have been manufactured, including plasma-derived (e.g., Immunine VH®, Mononine®) and recombinant FIX (e.g., BeneFIX®, Ixinity®, Rixubis®) (Mannucci and Franchini, 2014; Morfini and Zanon, 2016). Recombinant modified FIX versions such as PEGylated (Rebinyn®), Fc-fused (Alprolix®), and albumin-fused (Idelvion®) are highlighted due to their extended clearance time. Regarding the purification process, the chromatography strategies to purify these modified versions of FIX are compared in Figure 5.

Despite that different moieties are linked to FIX, all three modified FIX forms have one chromatographic step in common: an AEX step using chromatographic supports bearing quaternary amines. In all cases, a low ionic strength mobile phase with NaCl/CaCl_2_ was used to elute the modified FIX. FIX has a pI between 4.0 and 5.0 (Santagostino et al., 2012; Lee et al., 2014). Therefore, at neutral and basic pH values the protein can bind to the AEX support. The elution strategy exploits the structural properties of FIX. FIX is a vitamin K-dependent protein that requires calcium ions for proper biological activity. Once calcium ions are bound to FIX, the protein goes through conformational changes which can affect the surface charge (Yan, 1996; Blostein et al., 2008). Following this rationale, once the column has been loaded and the proteins have bound to the resin, a low CaCl_2_ concentration (50 mM) will detach FIX from the column by the loss of interactions caused by the conformational changes of the protein, while undesired proteins and contaminants will remain bound to the column.

The number of chromatographic steps in the downstream processing of modified therapeutic proteins will depend directly on the selected type of modification. The purification of PEGylated FIX requires only one chromatographic step (using POROS 50 HQ resin) because FIX is already pure prior to the PEGylation reaction. In contrast, the purification strategy of Alprolix® and Idelvion® requires a multi-step chromatographic approach, consisting of a capture step followed by two polishing steps. Both therapeutic proteins are produced in CHO cells and secreted to the culture medium and thus, a higher number of impurities must be removed.

In the case of Alprolix®, the capture step is focused in taking advantage of the high affinity of protein A ligand for the Fc region of the conjugate. Then, a weak AEX resin (diethylaminoethyl ligand) is employed to eliminate impurities and leached protein A. To achieve the required purity level, a second polishing step is applied with strong AEX (quaternary ammonium ligand) using the CaCl_2_ elution approach. The purification of Idelvion® involves Q Sepharose FF resin (Cytiva) as a capture step and CaCl_2_ for elution. IEX is commonly used for capture in part because of the high binding capacity that can be achieved. This feature, in addition to the FIX-selectivity of CaCl_2_ elution, makes it a suitable option for the capture step of Idelvion®. Afterwards, polishing steps are employed using heparin affinity ligand and CHT particles. Heparin ligand has affinity to coagulation factors but also to a broad range of other proteins; therefore, an additional purification step is necessary to ensure the complete removal of contaminant proteins and achieve the required purity. CHT particles have demonstrated high affinity and selectivity to native FIX; furthermore, the small particle size of CHT and their reduced pore size can provide a high resolution. In summary, the comparison of the purification processes of different modified FIX therapeutics revealed that structural properties of both, FIX and the conjugated moiety play a key role in the selection of the chromatographic techniques and the sequence to be employed. Additionally, the starting material (cell culture media or reaction buffer) must be considered to recognize the type and burden of impurities to be removed.

## Conclusions and Perspectives

Undoubtedly, given their superior pharmacokinetic and pharmacodynamic properties, more biopharmaceuticals based on modified therapeutic proteins will be developed and will reach the market in upcoming years. It is fair to say that due to the great impurity removal capacity of chromatography, this technique will continue to be the core of the downstream processing for modified therapeutic proteins. Therefore, the understanding of the rationale behind the selection of operating parameters for chromatographic processes (binding conditions, elution mode, ligand, resin, etc.) becomes crucial to increase the process yield, without compromising product purity.

The first aspect to consider is where the modified protein is being isolated from, which can be either a reaction mixture (starting with the pure protein) or a cell culture supernatant. This will determine the number and modes of chromatographic steps needed. Modified proteins from cell culture media (Fc-fusion, albumin-fusion, or glycosylation) may need up to four chromatographic steps; meanwhile PEGylated and lipidated proteins may only require one chromatographic step depending on the desired target product. As analyzed with the case study of coagulation factor IX, the moiety attached to the protein will determine the appropriate chromatographic modes to be employed and their sequence. It is essential to recognize or characterize the physicochemical properties of the native protein (e.g., hydrophobicity, surface charge, size, and affinity to certain ligands) and analyze which of these could be altered after conjugation in order to design a preliminary purification strategy.

Nowadays, novel stationary phases with enhanced properties are in development. Greater physical and chemical stability, high binding capacity, and high resolution will allow the operation of chromatographic columns at challenging industrial process conditions, such as high flow rates, while maintaining product recovery and purity. Among the new stationary phases with promising applications, MMC mode is highlighted since it provides versatility on ligand selection with high resolution in chromatography; although improvement of the backbone matrix of these resins is still needed to avoid non-specific interactions, increase lifetime, mechanical stability, and binding capacity. Despite that several resins with enhanced properties have been designed, produced, and tested, many of those chromatographic materials remain at experimental stages; probably because their application at large-scale have not achieved acceptable performances yet. Hence, most of the current purification processes rely on conventional, commercially available chromatographic supports. It is worthy to mention that all purification processes presented in this revision rely on the state of the art when all the biopharmaceuticals were developed. Therefore, it is feasible that all new chromatographic materials can be reported in more recently approved products.

Chromatographic operational parameters must be carefully thoroughly selected to attain the best possible performance with currently available resins. For all chromatographic modes, different elution conditions can be explored to optimize process performance, although this selection process is resource- and time-consuming. Fortunately, high throughput screening (HTS) methodologies are available, which can produce a massive amount of data through miniaturization and parallelization of experiments allowing the analysis of orthogonality and separability of resins. Combining the data generation power of HTS along with statistical tools, such as design of experiments, a much faster and guided process to establish customized, efficient, and cost-effective methodologies is obtained.

On the other hand, process integration, such as on-column PEGylation or lipidation, and continuous operations can also contribute to streamline the purification process and increase productivity. This approach, although still under development, is considered as a promising strategy since it reduces the number of unit operations required for the purification process and in consequence, cost and time are diminished.

Downstream processing continues to be the bottleneck of many biopharmaceutical manufacturing processes. Therefore, to the extent that this stage is truly optimized, the manufacturing process will flow more efficiently and satisfying the continuously increasing demand.
